# Galactose Inhibits the Translation of Erg1 that Enhances the Antifungal Activities of Azoles Against *Candida albicans*

**DOI:** 10.3390/antibiotics14080799

**Published:** 2025-08-05

**Authors:** Sijin Hang, Li Wang, Zhe Ji, Xuqing Shen, Xinyu Fang, Wanqian Li, Yuanying Jiang, Hui Lu

**Affiliations:** 1 Department of Pharmacy, Shanghai Tenth People’s Hospital, School of Medicine, Tongji University, Shanghai 200331, China; 2332388@tongji.edu.cn (S.H.);; 2 Key Laboratory of Pathogen-Host Interaction, Ministry of Education, School of Medicine, Tongji University, Shanghai 200331, China

**Keywords:** galactose, azoles, allylamines, translation of Erg1, *Candida albicans*

## Abstract

Background/Objectives: The diminished efficacy of azoles in treating fungal infections is attributed to the emergence of resistance among pathogenic fungi. Employing a synergistic approach with other compounds to enhance the antifungal activity of azoles has shown promise, yet the availability of clinically valuable adjuvants for azoles and allylamines remains limited. Studies have demonstrated that the human host environment provides multiple carbon sources, which can influence the susceptibility of *C. albicans* to antifungal agents. Therefore, a comprehensive investigation into the mechanisms by which carbon sources modulate the susceptibility of *C. albicans* to azoles may uncover a novel pathway for enhancing the antifungal efficacy of azoles. Methods: This study explored the impact of various carbon sources on the antifungal efficacy of azoles through methodologies including minimum inhibitory concentration (MIC) assessments, super-MIC growth (SMG) assays, disk diffusion tests, and spot assays. Additionally, the mechanism by which galactose augments the antifungal activity of azoles was investigated using a range of experimental approaches, such as gene knockout and overexpression techniques, quantitative real-time PCR (qRT-PCR), Western blot analysis, and cycloheximide (CHX) chase experiments. Results: This study observed that galactose enhances the efficacy of azoles against *C. albicans* by inhibiting the translation of Erg1. This results in the suppression of Erg1 protein levels and subsequent inhibition of ergosterol biosynthesis in *C. albicans*. Conclusions: In *C. albicans*, the translation of Erg1 is inhibited when galactose is utilized as a carbon source instead of glucose. This novel discovery of galactose’s inhibitory effect on Erg1 translation is expected to enhance the antifungal efficacy of azoles.

## 1. Introduction

Fungal infections pose a significant threat to human health, particularly among immunocompromised individuals [1,2]. The depletion of ergosterol leads to alterations in the lipid bilayer composition, which subsequently affects cell membrane fluidity and inhibits fungal growth [3]. As a result, the inhibition of ergosterol biosynthesis has become a well-established and effective strategy in antifungal therapy. Azoles, in particular, target lanosterol-14α-demethylase (referred to as Erg11 in *Candida albicans*) and disrupt ergosterol biosynthesis, thereby demonstrating clinical efficacy [4]. However, challenges persist, including issues related to toxicity, drug resistance, and the development of cross-resistance between agricultural and medical azoles [5,6]. The development of adjuvants to enhance the antifungal activity of azoles represents a promising approach to mitigate the onset of drug resistance in pathogenic fungi [7]. Despite this, few synergistic adjuvants for azoles are currently in clinical use. Therefore, there is an urgent need for novel strategies to enhance the antifungal efficacy of azoles [8].

*C. albicans*, a commensal fungal species, frequently colonizes the human gastrointestinal tract, where it encounters a diverse array of monosaccharides, including glucose, fructose, and galactose, as well as disaccharides such as sucrose and lactose [9]. In the bloodstream, where glucose is prevalent, *C. albicans* can penetrate and cause candidemia, subsequently infecting internal organs, which are characterized by lower glucose concentrations [10]. Research has demonstrated that carbon sources are critical to the growth and pathogenicity of *C. albicans*. For instance, enolase (referred to as Eno1 in *C. albicans*), which is involved in glycolysis and gluconeogenesis, is essential for both vegetative growth and hyphal development in *C. albicans*, as well as for its virulence in a murine infection model [11,12,13,14]. Similarly, kinases such as Hxk1, Hxk2, Glk1, and Glk4, which are implicated in sugar phosphorylation, are also significant for the virulence of *C. albicans* in a murine infection model [15]. Furthermore, carbon sources can influence the susceptibility of *C. albicans* to antifungal agents. For example, glucose can enhance the resistance of *C. albicans* to antifungal drugs such as miconazole and voriconazole [16,17]. Culturing *C. albicans* in lactate, as opposed to glucose, has been demonstrated to enhance the antifungal efficacy of fluconazole (FLC) and miconazole by diminishing ergosterol content. However, this alteration also results in increased resistance to amphotericin B and caspofungin [18,19,20]. Additionally, fructose has been shown to activate the efflux pumps Mdr1 and Cdr1, thereby inducing resistance to FLC in *C. albicans* [21]. Notably, elevated blood glucose levels have been recognized as a significant risk factor for the development of candidiasis, a potentially life-threatening complication in individuals with diabetes mellitus. Approximately 25% of patients with diabetes mellitus experience oral candidiasis [22,23,24]. Patients with Type I diabetes mellitus are more susceptible to mucosal and epidermal candidiasis, whereas those with Type II diabetes mellitus are more likely to develop invasive candidiasis [25,26]. The increase in serum glucose levels is a critical factor contributing to the heightened incidence of candidiasis among diabetic patients [25]. These observations suggest that carbon sources significantly influence the pathogenicity and drug susceptibility of *C. albicans*, although the precise mechanisms underlying these effects have yet to be fully elucidated.

Erg1, an enzyme associated with the endoplasmic reticulum (ER), is integral to the biosynthetic pathway of ergosterol, catalyzing the epoxidation of squalene to 2,3-oxidosqualene [27]. This enzyme is categorized as a class E monooxygenase, distinguished by its dependence on flavin adenosine dinucleotide (FAD) as a cofactor, which facilitates electron transfer from nicotinamide adenine dinucleotide phosphate (NADPH)-cytochrome P450 reductase to oxidize squalene and reduce molecular oxygen to water [28]. In the fungal pathogens *C. albicans* and *Candida glabrata* (also known as *Nakaseomyces glabrata*), downregulation of Erg1 expression is associated with decreased resistance to antifungal agents such as FLC, ketoconazole, terbinafine, and amphotericin B [29,30,31,32]. This observation suggests that Erg1 may be a promising target for the development of synergistic antifungal therapies. Furthermore, the transcription factor Upc2 in fungi is responsible for binding to sterol regulatory elements (SRE) within the promoters of genes involved in ergosterol biosynthesis, including the *ERG1* gene [33,34]. Research has demonstrated that the deletion of the *UPC2* gene increases the susceptibility of *C. albicans* to azole therapies, particularly in strains exhibiting high levels of azole resistance, due to the presence of multiple resistance mechanisms [35,36,37,38]. This finding highlights the potential efficacy of targeting the transcription of the *ERG1* gene to enhance the effectiveness of antifungal treatment. Furthermore, Erg1 undergoes ubiquitination by the Doa10 ubiquitin protein ligase, resulting in its degradation via the endoplasmic reticulum-associated degradation (ERAD) pathway [39]. The degradation of Erg1, prompted by lanosterol accumulation, has been observed in *Saccharomyces cerevisiae* cells treated with FLC [39]. Synergistic effects have been reported when combining terbinafine and azoles for the treatment of dermatophytosis [40,41], suggesting that promoting Erg1 degradation could be a viable strategy to enhance the efficacy of antifungal medications. Nonetheless, research into enhancing the antifungal activity of azoles, through the inhibition of Erg1 translation and reduction of its expression, remains limited.

In this study, we discovered that galactose, in contrast to glucose, can enhance the efficacy of azole antifungals against *C. albicans*. The synergistic effect of galactose is mediated through the inhibition of ergosterol biosynthesis in *C. albicans*. This inhibitory effect is associated with the suppression of Erg1 protein levels, achieved by inhibiting the translation of Erg1. These findings suggest that targeting the translation process of Erg1 with agents such as galactose could effectively reduce Erg1 protein levels and potentiate the antifungal efficacy of azoles against *C. albicans*.

## 2. Results

### 2.1. Galactose Enhanced the Antifungal Activities of Azoles Against C. albicans

**Figure 1:**
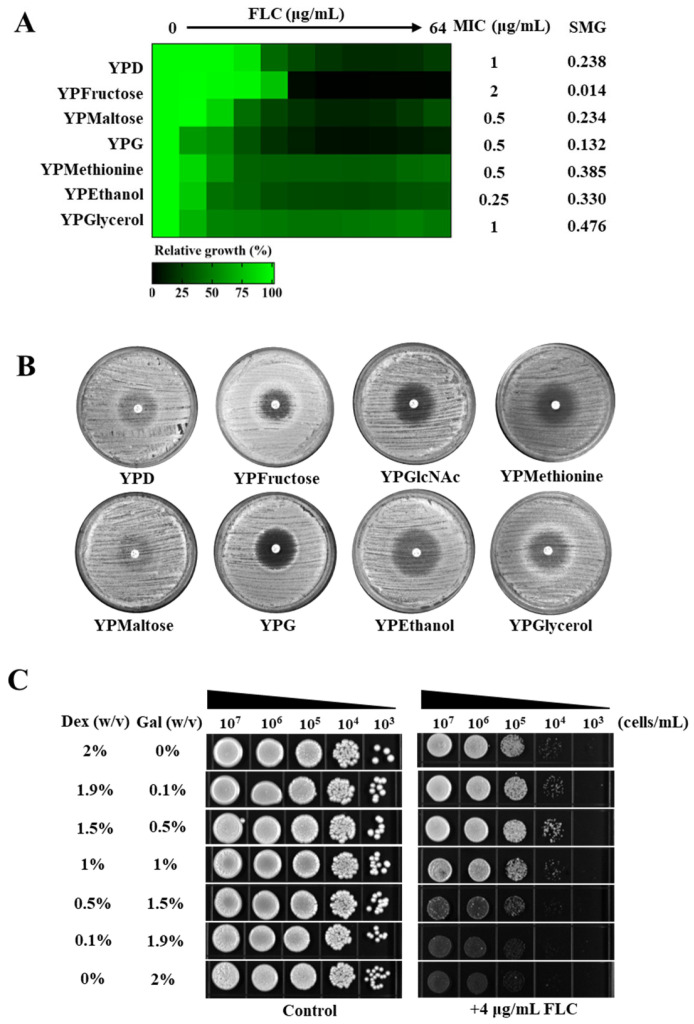
Galactose enhanced the antifungal activities of fluconazole (FLC) against *C. albicans.* (**A**) An assay to determine the minimum inhibitory concentration (MIC) and super-MIC growth (SMG) of FLC against *C. albicans* was conducted using media supplemented with various carbon sources. The medium designated as YPX consisted of yeast extract and bacteriological peptone, with the variable X representing the supplemented carbon sources. These carbon sources included dextrose (YPD), fructose (YPFructose), maltose (YPMaltose), galactose (YPG), methionine (YPMethionine), ethanol (YPEthanol), and glycerol (YPGlycerol). (**B**) A total of 1 × 10^6^ *C. albicans* cells were inoculated onto solid media plates containing YPD, YPFructose, YPMaltose, YPG, YPMethionine, YPEthanol, or YPGlycerol. Subsequently, an FLC disk, containing 25 μg of FLC, was placed at the center of each plate. The plates were then incubated at 30 °C for 48 h. (**C**) Spotting assays were conducted on solid YP medium supplemented with varying concentrations of dextrose (Dex) and galactose (Gal), specifically at the following Dex:Gal ratios: 2%:0%, 1.9%:0.1%, 1.5%:0.5%, 1%:1%, 0.5%:1.5%, 0.1%:1.9%, and 0%:1.9%. Media supplemented exclusively with dimethyl sulfoxide (DMSO) were utilized as control conditions.

To evaluate the influence of various carbon sources on the susceptibility of *C. albicans* to azoles, we conducted a minimum inhibitory concentration (MIC) assay using media supplemented with different carbon sources, including dextrose (YPD), fructose (YPFructose), maltose (YPMaltose), galactose (YPG), methionine (YPMethionine), ethanol (YPEthanol), and glycerol (YPGlycerol). The MIC values were utilized to determine the impact of these carbon sources on azole-resistant *C. albicans*. Additionally, super-MIC growth (SMG) values, calculated as the average growth per well above the MIC divided by the growth level in the absence of a azole, were used to assess the impact on azole tolerance [37,42]. Among the carbon sources tested, ethanol demonstrated the most pronounced effect in reducing the MIC value of FLC against *C. albicans*, decreasing from 1 μg/mL in YPD medium to 0.25 μg/mL in YPEthanol medium. However, ethanol had a minimal impact on the SMG value of FLC against *C. albicans*. In contrast, fructose exhibited the most pronounced reduction in the SMG value of FLC against *C. albicans*, decreasing from 0.238 in YPD medium to 0.014 in YPFructose medium. Nonetheless, fructose also led to an increase in the MIC value of FLC against *C. albicans*, increasing from 1 μg/mL in YPD medium to 2 μg/mL in YPFructose medium. In comparison to glucose, galactose reduced the MIC value of FLC against *C. albicans* from 1 μg/mL in YPD medium to 0.5 μg/mL in YPG medium and concurrently decreased the SMG value from 0.238 in YPD medium to 0.132 in YPG medium. Thus, when considering both the MIC and SMG parameters, galactose demonstrated the most potent synergistic effect with FLC among the carbon sources evaluated, relative to glucose (Figure 1A).

The findings were corroborated by a disk diffusion assay, in which the diameter of the inhibition zone reflects the resistance of *C. albicans* to FLC; a larger radius signifies reduced resistance. Additionally, the degree of clonal growth within the inhibition zone indicates the tolerance of *C. albicans* to FLC, with fewer colonies suggesting diminished tolerance. The assay results demonstrated that, among the carbon sources tested, the filter paper impregnated with 25 μg of FLC produced the largest clear zone of inhibition on the solid medium containing galactose as the carbon source (Figure 1B).

We examined the influence of galactose on the antifungal efficacy of FLC by modulating the concentration ratio of glucose to galactose in the culture medium. The results from spot assays indicated that progressively decreasing the glucose concentration from 2% (*w*/*v*) to 0% (*w*/*v*) while simultaneously increasing the galactose concentration from 0% (*w*/*v*) to 2% (*w*/*v*) did not impact the proliferation of *C. albicans*. However, an increase in the galactose concentration in the medium was associated with a marked enhancement in the sensitivity of *C. albicans* to FLC. Notably, when the glucose concentration was reduced to 0.5% (*w*/*v*), and the galactose concentration was increased to 1.5% (*w*/*v*), FLC (4 μg/mL) demonstrated significantly improved antifungal activity (Figure 1C).

Moreover, galactose significantly potentiates the efficacy of various azole antifungals, including miconazole, ketoconazole, itraconazole, and voriconazole, as well as that of the allylamine antifungal agent terbinafine (Table 1). Notably, galactose does not influence the antifungal activity of amphotericin B (targets plasma ergosterol) [43], caspofungin (targets Fks1) [44], 5-fluorocytosine (inhibits nucleic acid biosynthesis) [45], or menadione (induces reactive oxygen species generation) [46] (Table 1). This observation suggests that galactose specifically enhances the antifungal activities of azoles.

### 2.2. Galactose Enhanced the Antifungal Activity of Azoles Independent of the Activated Leloir Pathway

The environment significantly influences the interactome of Hsp90 in *C. albicans* and can be disrupted by external stressors [47]. The ongoing competition among client proteins for Hsp90 has been conceptualized as the “Hsp90 titration model”. This model posits that under specific environmental stress conditions, a substantial portion of Hsp90 is allocated to manage misfolded proteins, thereby reducing its availability for regular client proteins. As a result, wild-type cells exhibit phenotypes characterized by diminished levels of Hsp90 [48,49]. Upon exposure to galactose, *C. albicans* upregulates enzymes associated with the Leloir pathway, which metabolizes galactose, including galactokinase (Gal1), galactose-1-phosphate uridyl transferase (Gal7), and UDP-glucose 4-epimerase (Gal10) [50]. These enzymes are clients of Hsp90, and their upregulation intensifies competition for Hsp90 binding, thereby inhibiting its interaction with other clients, such as Ras1. The release of Ras1 from Hsp90 inhibition activates Ras1, which subsequently promotes mycelial formation. This mechanism elucidates how galactose facilitates mycelial development in *C. albicans* [48].

**Figure 2:**
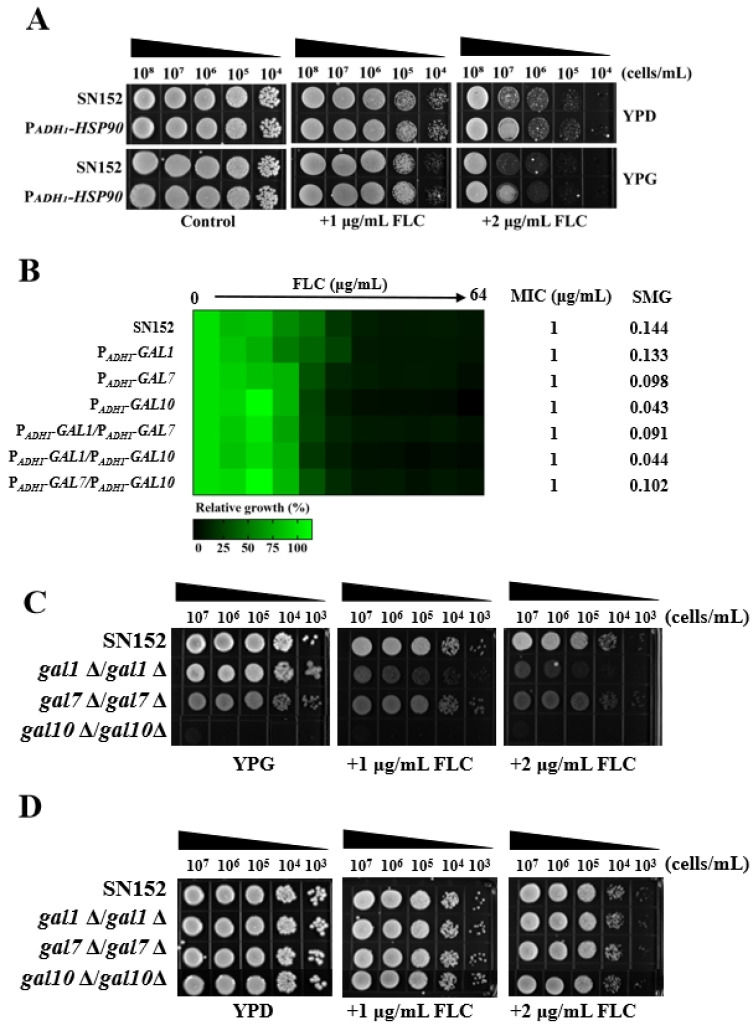
Galactose enhanced the antifungal activity of FLC independent of the activated Leloir pathway. (**A**) Spotting assays were conducted on solid YPD and YPG media with varying concentrations of FLC, utilizing the *C. albicans* wild-type strain SN152 and the *HSP90* gene overexpression mutant P*_ADH1_*-*HSP90*. YPD and YPG media supplemented exclusively with DMSO were utilized as control conditions. (**B**) An assay was performed to ascertain the MIC and SMG of FLC against *C. albicans.* This study utilized YPD medium and included the *C. albicans* wild-type strain SN152, as well as the overexpression mutants P*_ADH1_*-*GAL1*, P*_ADH1_*-*GAL7*, P*_ADH1_*-*GAL10*, P*_ADH1_*-*GAL1*/P*_ADH1_*-*GAL7*, P*_ADH1_*-*GAL1*/P*_ADH1_*-*GAL10*, and P*_ADH1_*-*GAL7*/P*_ADH1_*-*GAL10*. (**C**) Spotting assays were performed on solid YPG medium with different concentrations of FLC, employing the *C. albicans* wild-type strain SN152 and the gene deletion mutants *gal1*Δ/*gal1*Δ, *gal7*Δ/*gal7*Δ, and *gal*10Δ/*gal10*Δ. (**D**) Spotting assays were conducted on solid YPD medium containing varying concentrations of FLC, utilizing the *C. albicans* wild-type strain SN152 alongside the gene deletion mutants *gal1*Δ/*gal1*Δ, *gal7*Δ/*gal7*Δ, and *gal*10Δ/*gal10*Δ.

Research has established that Hsp90 is integral to the maintenance of azole tolerance and resistance in *C. albicans* [47,51] through several mechanisms, including its physical interaction with calcineurin [52,53], stabilization of Mkc1 signaling [54], modulation of ergosterol biosynthesis enzymes [47,55], and maintenance of Fks1 integrity [56]. It is postulated that galactose may inactivate Hsp90 client proteins involved in regulating *C. albicans* susceptibility to azoles, due to the upregulation of Gal1, Gal7, and Gal10 in the presence of galactose. If this hypothesis holds true, overexpression of the *HSP90* gene in *C. albicans* should provide sufficient Hsp90 to concurrently bind with proteins involved in the Leloir pathway and those associated with azole drug resistance, thereby negating the enhancement of azole antifungal activity by galactose. To test this hypothesis, we utilized the constitutively active *ADH1* promoter to overexpress the *HSP90* gene in the *C. albicans* strain SN152, resulting in the mutant strain P*_ADH1_*-*HSP90*. The overexpression of the *HSP90* gene led to increased resistance of *C. albicans* to FLC in both YPD and YPG media. However, the P*_ADH1_*-*HSP90* mutant exhibited heightened hypersensitivity to FLC in the YPG medium compared to the YPD medium (Figure 2A). This finding challenges the validity of the initial hypothesis.

If galactose enhances the antifungal activity of azoles independently of the activated Leloir pathway, then the overexpression of genes associated with the Leloir pathway in *C. albicans* is hypothesized to sequester more Hsp90. This sequestration could potentially inhibit the binding of Hsp90 to proteins associated with azole resistance, thereby enhancing the antifungal efficacy of FLC in glucose-based culture media. To test this hypothesis, we utilized the consistently highly expressed *ADH1* promoter to overexpress the genes *GAL1*, *GAL7*, *GAL10*, *GAL1* plus *GAL7*, *GAL1* plus *GAL10*, and *GAL7* plus *GAL10* in *C. albicans* SN152. This resulted in the creation of the mutants P*_ADH1_*-*GAL1*, P*_ADH1_*-*GAL7*, P*_ADH1_*-*GAL10*, P*_ADH1_*-*GAL1*/P*_ADH1_*-*GAL7*, P*_ADH1_*-*GAL1*/P*_ADH1_*-*GAL10*, and P*_ADH1_*-*GAL7*/P*_ADH1_*-*GAL10*. A comparative analysis with the wild-type strain SN152 revealed that these mutants exhibited similar susceptibility to FLC in YPD medium (Figure 2B). Consequently, this observation does not support the initial hypothesis.

Furthermore, if galactose enhances the antifungal activity of azoles independently of the activated Leloir pathway, then knocking out genes associated with the Leloir pathway in *C. albicans* will prevent the sequestration of Hsp90. This, in turn, will enhance the binding of Hsp90 to azole resistance-associated proteins, thereby diminishing the antifungal efficacy of FLC in glucose-based culture media. In contrast, when using galactose as the culture medium, the deletion of these genes does not affect the antifungal activity of FLC. To test this hypothesis, we knocked out the *GAL1*, *GAL7*, and *GAL10* genes in *C. albicans*, resulting in the mutants *gal1*Δ/*gal1*Δ, *gal7*Δ/*gal7*Δ, and *gal10*Δ/*gal10*Δ. We observed that the absence of the *GAL10* gene rendered *C. albicans* nonviable in YPG medium, while the loss of the *GAL1* and *GAL7* genes increased the susceptibility of *C. albicans* to FLC in YPG medium (Figure 2C). However, in YPD medium, the sensitivity of the mutant strains *gal1*Δ/*gal1*Δ, *gal7*Δ/*gal7*Δ, and *gal10*Δ/*gal10*Δ to FLC was comparable to that of the wild-type strain SN152 (Figure 2D). Collectively, these findings indicate that galactose enhances the antifungal activity of FLC independently of the activated Leloir pathway.

### 2.3. Galactose Inhibits Ergosterol Biosynthesis in C. albicans

**Figure 3:**
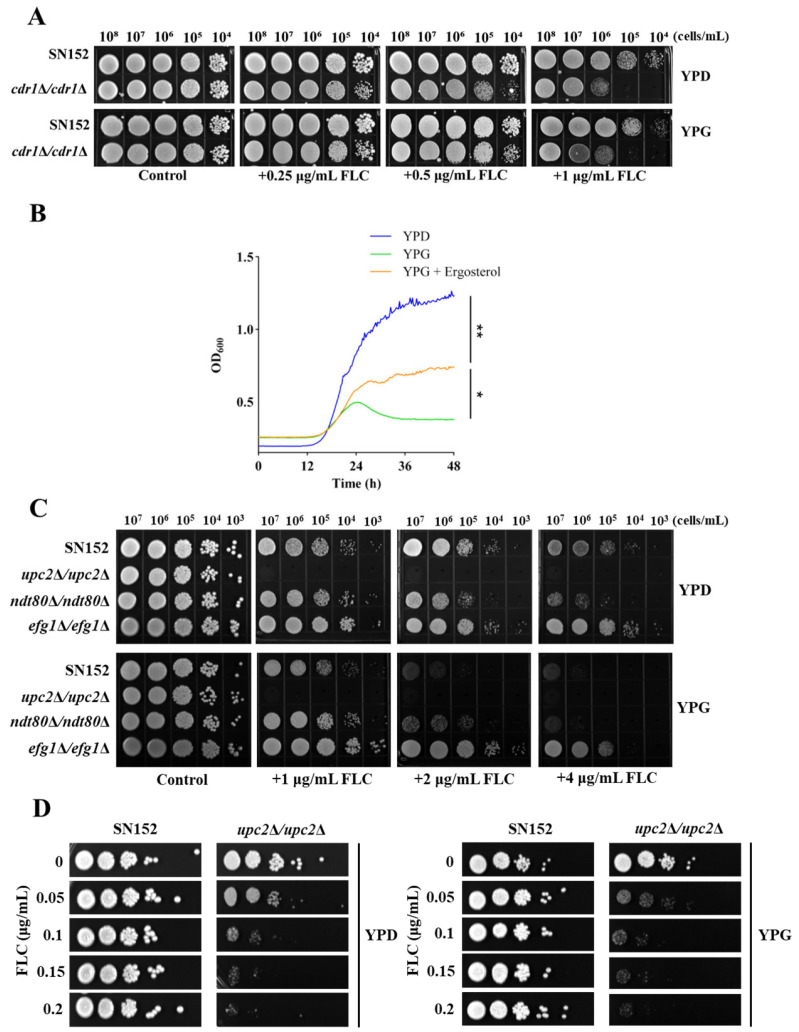
Galactose inhibits ergosterol biosynthesis in *C. albicans*. (**A**) Spotting assays were performed on solid YPD and YPG media containing different concentrations of FLC, using the *C. albicans* wild-type strain SN152 and the *CDR1* gene deletion mutant *cdr1*Δ/*cdr1*Δ. Media supplemented solely with DMSO served as control conditions. (**B**) For the growth inhibition curve assays, *C. albicans* (strain SN152) cells were cultivated in either YPD medium, YPG medium, or YPG medium supplemented with 100 μM ergosterol (designated as YPG + Ergosterol). * *p* < 0.05; ** *p* < 0.01. (**C**) Spotting assays were conducted on solid YPD and YPG media containing varying concentrations of FLC, utilizing the *C. albicans* wild-type strain SN152, alongside the gene deletion mutants *upc2*Δ/*upc2*Δ, *ndt80*Δ/*ndt80*Δ, and *efg1*Δ/*efg1*Δ. Media supplemented solely with DMSO were used as control conditions. (**D**) Spotting assays were conducted on solid YPD and YPG media containing varying concentrations of FLC, utilizing the *C. albicans* wild-type strain SN152 and the *upc2*Δ/*upc2*Δ mutant.

There are potentially two primary mechanisms through which galactose enhances the antifungal efficacy of azoles: augmentation of intracellular drug concentration and reduction of intracellular ergosterol content. If galactose were to increase the intracellular concentration of the drug, the deletion of Cdr1, a key protein involved in azole efflux [57], would negate the variations in *C. albicans* susceptibility to azoles induced by different carbon sources. However, our findings indicate that galactose does not operate through this mechanism, as the absence of Cdr1 did not alter the enhancement of antifungal activity of FLC by galactose (Figure 3A). In comparison to a glucose-based culture medium (YPD medium), *C. albicans* exhibits slower growth in a galactose-based medium (YPG medium). Nevertheless, the addition of ergosterol (100 μM) facilitates the growth of *C. albicans* in the YPG medium, suggesting that galactose reduces the intracellular ergosterol content in *C. albicans* (Figure 3B). These results imply that galactose diminishes the intracellular ergosterol content in *C. albicans*.

A potential mechanism by which galactose inhibits ergosterol synthesis is through the suppression of gene expression associated with this pathway. To explore this hypothesis, we deleted three critical transcriptional regulatory factors—Upc2, Efg1, and Ndt80—that are known to modulate the expression of genes involved in ergosterol synthesis. We then assessed the effect of these gene deletions on the susceptibility of *C. albicans* to FLC [33,58,59]. Notably, in comparison to the YPD medium, FLC exhibited enhanced antifungal efficacy against the *upc2*Δ/*upc2*Δ, *efg1*Δ/*efg1*Δ, and *ndt80*Δ/*ndt80*Δ null mutants in the YPG medium (Figure 3C,D). These findings suggest that the reduction in intracellular ergosterol levels in *C. albicans* by galactose is not attributable to the inhibition of transcriptional activity of these regulatory factors.

### 2.4. Galactose Suppresses the Expression of Erg1 in C. albicans

**Figure 4:**
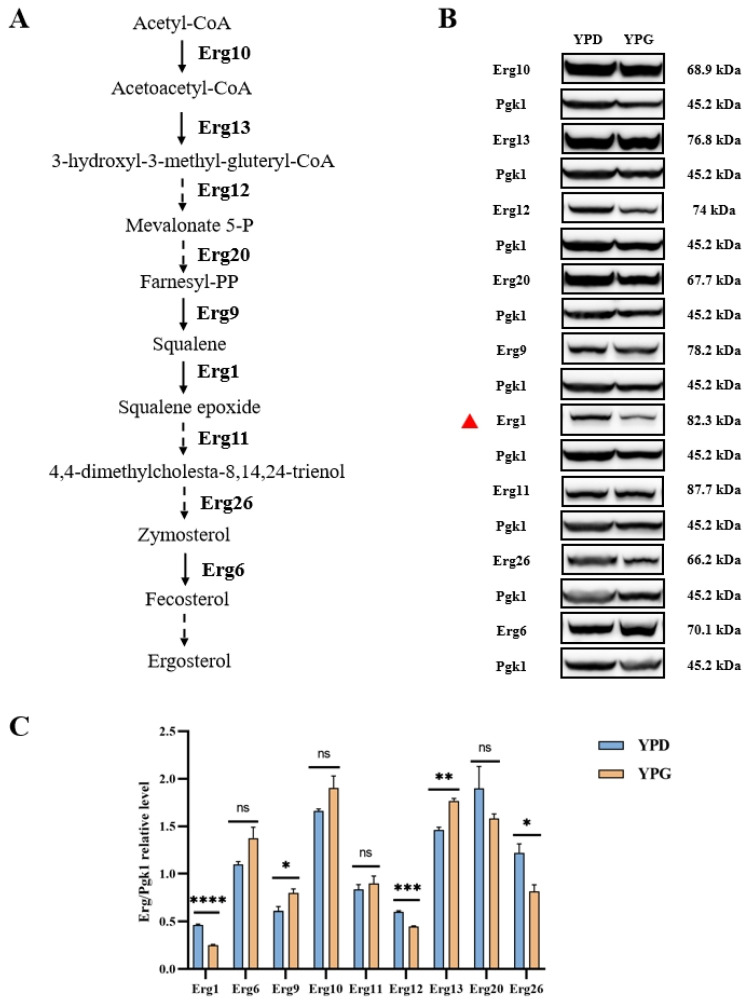
Galactose suppresses the expression of Erg1 in *C. albicans*. (**A**) A schematic representation of the ergosterol biosynthesis pathway in *C. albicans*. (**B**) When *C. albicans* mutants Erg1-GFP, Erg6-GFP, Erg9-GFP, Erg10-GFP, Erg11-GFP, Erg12-GFP, Erg13-GFP, Erg20-GFP, and Erg26-GFP were cultured in YPD and YPG media, an immunoblot analysis was performed to assess the expression of Erg1, Erg6, Erg9, Erg10, Erg11, Erg12, Erg13, Erg20, and Erg26. The red triangle denotes the protein exhibiting the most pronounced differential expression between the two culture media. (**C**) Quantification of the signal intensity ratio of Erg-GFP to Pgk1. ns, not significant; * *p* < 0.05; ** *p* < 0.01; *** *p* < 0.001; **** *p* < 0.0001.

Galactose may reduce the levels of enzymes associated with ergosterol synthesis, thereby decreasing intracellular ergosterol concentrations. To investigate this hypothesis, we tagged the key C-terminal regions involved in ergosterol synthesis with green fluorescent protein (GFP), focusing on enzymes such as Erg1, Erg6, Erg9, Erg10, Erg11, Erg12, Erg13, Erg20, and Erg26 [51], all of which play roles in ergosterol biosynthesis (Figure 4A). Our results demonstrate that galactose significantly diminishes the protein levels of Erg1 in YPG medium compared to YPD medium (Figure 4B,C). These findings suggest that galactose induces a reduction in intracellular ergosterol levels by decreasing the protein expression of Erg1.

### 2.5. Galactose Inhibits the Translation of Erg1 in C. albicans

**Figure 5:**
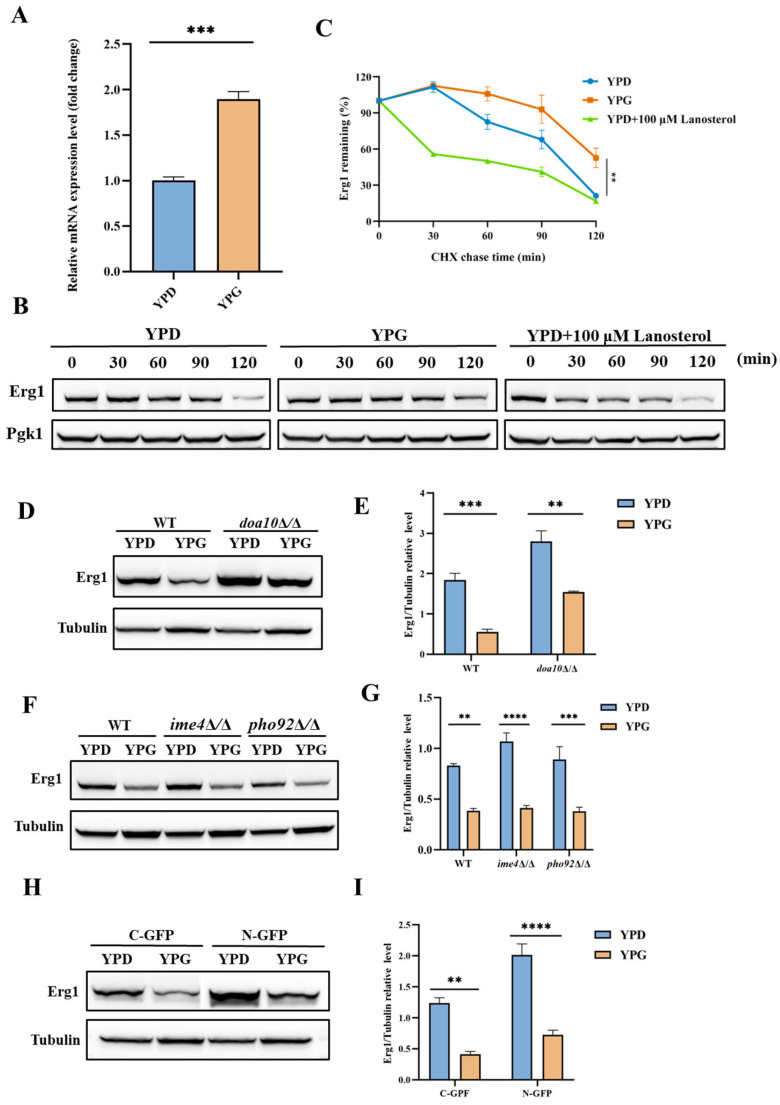
Galactose inhibits the translation of Erg1 in *C. albicans*. (**A**) When the *C. albicans* wild-type strain SN152 was cultured in either YPD medium or YPG medium, a quantitative real-time polymerase chain reaction (qRT-PCR) was conducted to evaluate the mRNA level of the *ERG1* gene. *** *p* < 0.001. (**B**) When *C. albicans* mutant Erg1-GFP was cultured in either YPD medium, YPG medium, or YPG medium supplemented with 100 μM lanosterol, an cycloheximide (CHX) chase analysis was conducted. (**C**) Quantification of the degradation of Erg1-GFP calculated as the ratio of Erg1-GFP to Pgk1. ** *p* < 0.01. (**D**) When *C. albicans* mutants Erg1-GFP and Erg1-GFP:: *doa10*Δ/Δ (in which the *DOA10* gene was deleted in the Erg1-GFP mutant) were cultured in YPD and YPG media, an immunoblot analysis of Erg1 was conducted. (**E**) Quantification of the signal intensity ratio of Erg1-GFP to Tubulin. ** *p* < 0.01; *** *p* < 0.001. (**F**) When *C. albicans* mutants Erg1-GFP, Erg1-GFP:: *ime4*Δ/Δ (in which the *IME4* gene was deleted in the Erg1-GFP mutant) and Erg1-GFP:: *pho92*Δ/Δ (in which the *PHO92* gene was deleted in the Erg1-GFP mutant) were cultured in YPD and YPG media, an immunoblot analysis of Erg1 was conducted. (**G**) Quantification of the signal intensity ratio of Erg1-GFP to Tubulin. ** *p* < 0.01; *** *p* < 0.001; **** *p* < 0.0001. (**H**) When *C. albicans* mutants Erg1-GFP (C-GFP) and GFP-Erg1 (N-GFP) were cultured in YPD and YPG media, an immunoblot analysis of Erg1 was conducted. (**I**) Quantification of the signal intensity ratio of Erg1-GFP to Tubulin. ** *p* < 0.01; **** *p* < 0.0001.

Galactose has the potential to reduce Erg1 protein expression in *C. albicans* cells by inhibiting the *ERG1* gene transcription, suppressing mRNA translation of the *ERG1* gene, or promoting Erg1 degradation. In our study, we employed quantitative reverse transcription PCR (qRT-PCR) to evaluate the mRNA levels of the *ERG1* gene. Notably, exposure of *C. albicans* to galactose, as opposed to glucose, resulted in a significant increase in *ERG1* mRNA levels (Figure 5A). As previously discussed, the reduction in intracellular ergosterol levels in *C. albicans* upon galactose exposure is not due to the inhibition of transcriptional activity of regulatory factors such as Upc2. Therefore, it can be inferred that the decreased Erg1 protein levels in *C. albicans* exposed to galactose are not attributable to reduced mRNA levels of the *ERG1* gene. Instead, galactose appears to impair Erg1 protein levels and subsequently elevate the mRNA levels of the *ERG1* gene through a feedback mechanism.

Cycloheximide (CHX) chase analysis was utilized to evaluate the degradation rates of Erg1 in *C. albicans* when exposed to glucose and galactose. In alignment with previous studies [39], our CHX chase analysis demonstrated that lanosterol significantly accelerated the degradation of Erg1 in the presence of glucose (Figure 5B,C). In contrast, exposure to galactose, as compared to glucose, did not enhance the degradation of Erg1 and may have even prolonged its stability (Figure 5B,C).

Erg1 has been identified as a substrate for ubiquitination by the Doa10 ubiquitin protein ligase, leading to its subsequent degradation via the ERAD pathway [39]. In the *C. albicans* Erg1-GFP mutant, deletion of the *DOA10* gene resulted in the accumulation of Erg1 in the *doa10*Δ/*doa10*Δ null mutant, as demonstrated by Western blot analysis, in comparison to the wild-type strain under galactose exposure. Notably, the *doa10*Δ/*doa10*Δ null mutant displayed reduced levels of Erg1 in YPG medium relative to YPD medium (Figure 5D,E). These observations indicate that, while the degradation of Erg1 is inhibited, the presence of galactose nonetheless results in a decrease in Erg1 concentration. This implies that galactose may inhibit the synthesis of Erg1. Given that galactose increases the mRNA levels of the *ERG1* gene, it is plausible to hypothesize that galactose suppresses the mRNA translation of the *ERG1* gene in *C. albicans*.

The influence of methylation modifications on mRNA has been shown to affect protein translation [60]. Utilizing a sequence-based N6-methyladenosine (m6A) modification site predictor (http://www.cuilab.cn/sramp/, (accessed on 1 December 2024)) [61], we identified potential m6A sites within the mRNA sequence of the ERG1 gene, suggesting the presence of m6A modifications in this mRNA. To investigate the potential role of galactose in inhibiting the translation of Erg1 through the regulation of m6A modification of the *ERG1* gene mRNA, we performed genetic deletions of the IME4 gene (encoding mRNA N6-adenosine methyltransferase) and the C5_01300C_A gene (encoding an N6-methyladenosine-containing RNA reader, which is an ortholog of *S. cerevisiae* Pho92) in the Erg1-GFP mutant. Western blot analysis revealed that the loss of *IME4* and C5_01300C_A genes in *C. albicans* did not prevent galactose from decreasing the expression of Erg1 (Figure 5F,G), indicating that the translational inhibition of Erg1 by galactose is independent of m6A modifications.

Previous studies have demonstrated that the *ERG1* gene mRNA, which contains potential AU-rich elements (AREs) within its 3′ untranslated region (UTR), shows elevated levels in iron-deficient cells lacking the RNA-binding proteins Cth1 and Cth2 in *S. cerevisiae* [62,63,64]. To examine whether galactose influences the translation of Erg1 by modulating its 3′UTR or possibly 5′UTR, the C-terminus and N-terminus of Erg1 were tagged with GFP, leading to the disruption of *ERG1* mRNA and its 3′UTR, or potentially the 5′UTR with GFP and the auxotrophic gene mRNA. Nonetheless, Western blot analysis indicated that galactose suppressed Erg1 expression in both Erg1-GFP and GFP-Erg1 mutants (Figure 5H,I), suggesting that galactose inhibits Erg1 translation without affecting the 3′UTR or 5′UTR of the *ERG1* gene mRNA.

## 3. Discussion

The biosynthesis of ergosterol in *C. albicans* is characterized by three distinct stages. Initially, acetyl-CoA is converted into mevalonate within the vacuole and mitochondria. The second stage culminates in the synthesis of farnesyl pyrophosphate in the vacuole. The third stage occurs on the ER membrane, where squalene synthase catalyzes the conversion of farnesyl pyrophosphate into squalene. Subsequently, squalene is transformed into lanosterol through the actions of squalene epoxidase and lanosterol synthase. Lanosterol undergoes further modifications to become ergosterol via enzymatic reactions facilitated by Erg11, Erg24, and the Erg251-Erg26-Erg27 complex. Importantly, when azoles inhibit Erg11, an alternative sterol synthesis pathway is activated. This pathway involves C-4 sterol methyl oxidase (Erg251), C-3 sterol dehydrogenase (Erg26), 3-keto sterol reductase (Erg27), and C-5 sterol desaturase (Erg3), resulting in the production of 14α-methylsterols. These 14α-methylsterols can functionally replace ergosterol in the presence of azole drugs, thereby imparting azole tolerance to *C. albicans* [37]. Research findings suggest that targeting ergosterol synthases can further inhibit ergosterol biosynthesis, thereby significantly enhancing the antifungal efficacy of azoles [8]. The concomitant use of these drugs with azoles can reduce ergosterol levels in fungal cells and augment the antifungal effect of azoles by inhibiting various synthases and altering ergosterol intermediate levels. Pitavastatin, a statin, inhibits Hmg1, thereby enhancing the activity of FLC against azole-resistant *Candida* and conferring fungicidal properties to FLC [65]. Nevertheless, statins may induce adverse effects and drug interactions [66]. Zoledronic acid, a treatment for osteoporosis, inhibits Erg20, thereby enhancing the activity of azoles against *Cryptococcus* and reducing resistance [67]. However, its affinity for bone and the associated risk of osteonecrosis limit its application in the treatment of invasive fungal diseases [68]. The small molecule CZ66 inhibits Erg251, suppressing 14α-methylsterol synthesis and causing the accumulation of toxic sterols, which overcomes azole tolerance in *C. albicans* and enhances the antifungal activity of azoles [37]. Nonetheless, CZ66 exhibits a short half-life in mice, which does not significantly enhance the in vivo antifungal effect of azoles [37]. Terbinafine, an Erg1 inhibitor, exhibits substantial antifungal efficacy against *Aspergillus*, azole-resistant *Candida*, and dermatophytes when administered in conjunction with azoles [69,70]. Nevertheless, the rapid development of resistance by pathogenic fungi to terbinafine constrains its clinical utility in therapeutic applications [71]. This study discovered that galactose, a fermentation-derived carbon source, inhibits the translation process of Erg1, leading to a reduction in Erg1 protein levels and subsequent inhibition of ergosterol biosynthesis, thereby enhancing the antifungal activity of FLC. These findings offer novel insights into augmenting the antifungal efficacy of azoles.

The synthesis of ergosterol is modulated by a variety of factors, notably the availability of carbon sources. The influence of carbon sources on ergosterol synthesis is attributable to their provision of essential precursors and energy required for the biosynthetic pathway. Variations in carbon sources can result in differential metabolic flux through the ergosterol biosynthesis pathway, thereby impacting ergosterol levels. This phenomenon is particularly significant in the context of fungal growth and development, as well as in the organism’s response to antifungal agents [72]. In the current study, it was observed that galactose enhances the efficacy of azoles against *C. albicans* by suppressing the translation of Erg1, reducing Erg1 protein levels, and ultimately inhibiting ergosterol biosynthesis. These findings suggest that carbon sources can regulate ergosterol synthesis not only through alterations in energy metabolism but also by specifically modulating the levels of enzymes involved in ergosterol biosynthesis, such as Erg1, akin to the effects observed with galactose. Unfortunately, this study did not clarify the precise molecular mechanism through which galactose inhibits Erg1 translation, necessitating further investigation in future experiments.

## 4. Materials and Methods

### 4.1. Strains, Primers, Agents, and Cultural Conditions

All strains and primers utilized in this study are detailed in Supplementary Tables S1 and S2, respectively. *C. albicans* strains were cultured overnight at 30 °C in YPD medium (comprising 1% yeast extract (OXOID Ltd., Basingstoke, UK), 2% bacteriological peptone (OXOID Ltd., Basingstoke, UK), and 2% dextrose (purity ≥ 99.5%) (Sangon Biotech, Shanghai, China) or YPG medium (comprising 1% yeast extract, 2% bacteriological peptone, and 2% galactose (purity ≥ 98.0%)) (Sangon Biotech, Shanghai, China). To analyze the effects of various carbon sources on the efficacy of antifungal drugs against *C. albicans*, YP medium (comprising 1% yeast extract and 2% bacteriological peptone) was supplemented with 2% concentrations of fructose (purity ≥ 99%)(Sangon Biotech, Shanghai, China), maltose monohydrate (purity ≥ 98%) (Aladdin, Shanghai, China), methionine (purity 98.5~101.5%)(Sangon Biotech, Shanghai, China), ethanol (purity ≥ 99.7%) (Sangon Biotech, Shanghai, China), or glycerol (purity ≥ 99%) (Sangon Biotech, Shanghai, China). The solid medium was fortified with 2% agar. Storage solutions for various antifungal agents, including FLC, miconazole, ketoconazole, voriconazole, itraconazole, amphotericin B, terbinafine, caspofungin, 5-Fluorocytidine, and menadione, all at a concentration of 6.4 mg/mL (TargetMol, Boston, MA, USA), were prepared using dimethyl sulfoxide (DMSO) as the solvent. The storage solution for ergosterol (10 mM) from Sangon Biotech, Shanghai, China, was prepared using a 50% Tween 80:50% ethanol mixture, while lanosterol (10 mM) from MCE, Shanghai, China, was dissolved in absolute ethanol.

### 4.2. MIC and SMG Assay

The MIC and SMG assays were conducted following previously established protocols [37]. The drug concentrations were prepared using the serial dilution method. *C. albicans* cells were seeded at a density of 1 × 10^3^ cells/mL, with 100 μL per well in a 96-well plate. For the MIC assay, the cells were incubated for 24 h at 30 °C, whereas for the SMG assay, the incubation period was extended to 48 h at the same temperature. Optical densities were measured at an absorbance of 600 nm using a Multiskan Sky spectrophotometer (ThermoFisher Scientific, Singapore). The MIC of the compound against *C. albicans* was established as the concentration necessary to achieve a 50% reduction in cell growth compared to the control. The SMG value was defined as the average growth per well above the MIC, divided by the growth level in the absence of the drug. The experiments were conducted with three replicates.

### 4.3. Disk Diffusion Assay

The disk diffusion assay was performed following the protocol described in a previous study [65]. After incubating *C. albicans* at 30 °C for 16 h, the cells were diluted with phosphate-buffered saline (PBS) buffer (136.89 mM NaCl, 2.67 mM KCl, 8.1 mM Na_2_HPO_4_, and 1.76 mM KH_2_PO_4_) to a concentration of 1 × 10^6^ cells/mL. A disposable cotton swab was used to apply the yeast solution onto a solid medium. A paper disc containing 25 µg of FLC was placed at the center of the dish. The dishes were then incubated at 30 °C for 48 h. The presence of a clear inhibition zone was used as an indicator of the susceptibility of *C. albicans* to FLC, assessed by observing the edge of the inhibition zone.

### 4.4. Spotting Assay

The spotting assay was conducted in accordance with the protocol outlined in a previous study [73]. Overnight cultures of *C. albicans* were diluted at a 1:10 ratio using PBS buffer. Subsequently, 3 μL aliquots of the diluted *C. albicans* cells were systematically spotted onto YPD agar plates. The cell concentrations utilized were 1 × 10^7^, 1 × 10^6^, 1 × 10^5^, 1 × 10^4^, and 1 × 10^3^ cells/mL. The plates were incubated at 30 °C for 48 h, followed by photographic documentation and subsequent analysis.

### 4.5. Growth Inhibition Curve Assay

The growth inhibition curve assay was conducted following the methodology outlined in a previous study [38]. *C. albicans* was inoculated at a concentration of 1 × 10^3^ cells/mL in either YPD medium, YPG medium, or YPG medium supplemented with 100 μM ergosterol and subsequently dispensed into 96-well plates at a volume of 150 μL per well. The plates were incubated for 48 h at 30 °C with continuous shaking. Optical density measurements at 600 nm (OD600) were recorded every 15 min over the 48 h period using a Tecan plate reader (Infinite 200 PRO, Grödig, Austria).

### 4.6. Disruption of Target Genes

Fusion PCR was employed to generate homologous recombinant DNA fragments designed to disrupt the target gene [74]. These fusion DNA fragments comprised the upstream and downstream sequences of the target gene, along with a selectable trophic marker, either *HIS1* or *ARG4*. Specifically, primers P1 and P3 were utilized to amplify the left flanking sequences, while primers P4 and P4 were used for the right flanking sequences of the target gene, with SN152 genomic DNA serving as the template. Additionally, universal primers P2 and P5 were employed to amplify the selectable *HIS1* or *ARG4* marker, using plasmids pSN52 or pSN69 as templates. Subsequently, a fusion product was synthesized using primers P1 and P6, with the three PCR products serving as templates, which was then introduced into SN152 strains. Strains containing the fusion segments were screened using media supplemented with the necessary auxotrophic supplements, followed by PCR verification.

### 4.7. Ectopic Overexpression of Target Genes

In accordance with the previously outlined methodology [75], the target gene was inserted into the pCPC18 vector to achieve ectopic overexpression. Initially, primers F1 and R1 facilitated the amplification of the target gene through the first round of PCR, resulting in a product characterized by a 15-base pair flanking homology region. Subsequently, this product was assembled with the vector using ligation-independent cloning techniques. Following transformation and integration, the target gene was incorporated into the *ADE2* locus, and its expression was regulated by the constitutive *ADH1* promoter.

### 4.8. C-Terminal or N-Terminal of Proteins Tagging GFP

To achieve C-terminal tagging of proteins with GFP, a two-step PCR method was utilized to amplify the plasmid pCPC64, thereby generating the desired DNA cassettes for GFP tagging. In the initial PCR round, primers F1 and R1 were employed to produce a product containing a 39 bp homology region. This product then served as the template for a second PCR round using primers F2 and R2, resulting in a 78 bp homologous DNA fragment of the target gene. The final PCR product was introduced into the SN152 strain via homologous recombination, facilitating the integration of the GFP tag at the target gene’s C-terminal. For N-terminal GFP tagging, the plasmid pCPC158 was amplified. This process also involved two rounds of PCR to generate DNA cassettes with 78 bp homology regions to the target gene, using primers F1 and R1 in the first round and F2 and R2 in the second. The resultant product was transformed into the SN152 strain to produce a mutant strain with the target gene’s N-terminal tagged with GFP [75].

### 4.9. RNA Extractions and Quantitative RT-PCR

*C. albicans* was initially cultured in 10 mL of YPD medium for 24 h at 30 °C. Subsequently, a 1:100 dilution was transferred into 100 mL of fresh YPD or YPG medium and incubated for an additional 16 h. Total RNA was extracted utilizing the YeaStar RNA Kit (ZymoResearch, Tustin, CA, USA). Reverse transcription of the isolated RNA was conducted using the PrimeScript™RT reagent Kit (Takara Bio, Kusatsu, Japan). Quantification of cDNA was performed using the TB Green^®^ Premix DimerEraser™ (Takara Bio, Shiga, Japan) on a Roche LightCycler^®^ 96 system (Roche, Carlsbad, CA, USA). The relative expression levels of the target gene were normalized against the reference *ACT1* gene. *ACT1* was one of the most table reference genes for normalization, with a stable expression level in *Candida* species [76]. Data interpretation was carried out using the 2^−ΔΔCt^ method, with results expressed as fold changes relative to the untreated control [77].

### 4.10. Western Blotting

*C. albicans* was initially cultured in 10 mL of YPD medium for 24 h at 30 °C. Subsequently, a 1:100 dilution was transferred to 100 mL of either YPD or YPG medium and incubated for an additional 16 h. The cells were harvested by centrifugation and resuspended in 500 μL of lysis buffer, which consisted of 5 mM ethylene diamine tetraacetic acid (EDTA) (pH 8.0), 1 mM phenylmethylsulfonyl fluoride (PMSF), and 1.0% Protease Inhibitor Cocktail (TargetMol, Boston, MA, USA). Cell lysis was performed using glass beads in a Beadruptor12 grinder (OMNI International, Kennesaw, GA, USA). The lysate was then centrifuged at 5000× *g* for 2 min at 4 °C, and the supernatant, containing the total protein extract, was collected. This protein solution was subjected to 4–20% sodium dodecyl sulfate (SDS)–polyacrylamide gel electrophoresis (PAGE) and subsequently transferred onto a polyvinylidene difluoride (PVDF) membrane. After blocking, the membrane was incubated with GFP (B-2) antibody (Santa Cruz Biotechnology, Santa Cruz, CA, USA, sc-9996) for 2 h at room temperature. Detection was carried out using a secondary antibody, goat anti-mouse IgG-HRP (Santa Cruz, sc-516102), and the ECL Western blotting kit (Epizyme, Shanghai, China) and photographed using the Tanon 5200 Chemiluminescence Imaging System (Tanon, Shanghai, China).

### 4.11. CHX Chase Analysis

*C. albicans* was cultured for 16 h, after which the yeast concentration was adjusted to an optical density at OD600 of 0.2. The culture was then incubated at 30 °C until reaching an OD600 of 1. Subsequently, the yeast cells were harvested by centrifugation at room temperature (5000× *g* for 2 min). Each 10 mL aliquot of the yeast suspension was resuspended in 2.5 mL of fresh YPD or YPG medium. CHX (1 mg/kg; TargetMol, Boston, MA, USA) was immediately added to initiate the treatment, and the timing was recorded. At each designated time point, 10 mL of the suspension was collected for protein extraction [78]. The positive control group was treated with 100 μM lanosterol (Sangon Biotech, Shanghai, China).

### 4.12. Statistical Analysis

All experiments were conducted in triplicate, and data were expressed as mean ± standard error of the mean (SEM). Data analysis was performed using GraphPad Prism version 9.0. For statistical evaluation, an ordinary one-way analysis of variance (ANOVA) followed by Dunnett’s multiple comparisons test, or a two-way ANOVA followed by Sidak’s multiple comparisons test, were employed. Statistical significance was denoted by *p* values as follows: not significant (ns); * *p* < 0.05; ** *p* < 0.01; *** *p* < 0.001; **** *p* < 0.0001.

## 5. Conclusions

In the present study, it was observed that galactose, unlike the preferred carbon source glucose for *C. albicans*, can enhance the efficacy of azole antifungals against this organism. The synergistic effect of galactose was found to be mediated through the inhibition of ergosterol biosynthesis in *C. albicans*. Notably, this inhibition is independent of the upregulation of Leloir pathway proteins (Gal1, Gal7, and Gal10) via competition for Hsp90. Instead, it is associated with the suppression of Erg1 protein levels. The finding that galactose reduces Erg1 protein expression by inhibiting its translation, rather than affecting *ERG1* gene transcription or promoting Erg1 degradation, indicates that targeting the translation process of Erg1 with agents such as galactose could effectively decrease Erg1 protein levels and, consequently, impede ergosterol synthesis. This strategy may enhance the antifungal efficacy of azoles against *C. albicans*.

## Figures and Tables

**Table 1:** The minimum inhibitory concentration (MIC) values of antifungal agents against *C. albicans* were assessed in media supplemented with dextrose (YPD) and galactose (YPG).

Compounds	Structures	MIC in YPD Medium	MIC in YPG Medium
Fluconazole	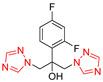	1 μg/mL	0.5 μg/mL
Miconazole	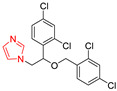	0.063 μg/mL	0.031 μg/mL
Ketoconazole	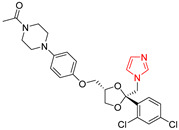	0.031 μg/mL	0.016 μg/mL
Voriconazole	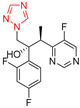	0.016 μg/mL	0.008 μg/mL
Itraconazole	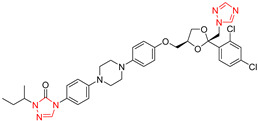	0.008 μg/mL	0.004 μg/mL
Terbinafine	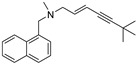	2 μg/mL	0.5 μg/mL
Amphotericin B	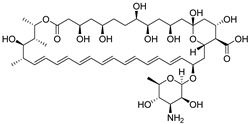	0.5 μg/mL	0.5 μg/mL
Caspofungin	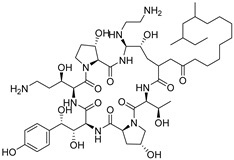	40 ng/mL	40 ng/mL
5-Fluorocytosine	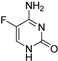	2 μg/mL	2 μg/mL
Menadione	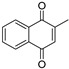	8 μg/mL	8 μg/mL

## Data Availability

Data are contained within the article and its Supplementary Materials.

## Supplementary Materials

The following supporting information can be downloaded at https://www.mdpi.com/article/10.3390/antibiotics14080799/s1. Supplementary Table S1: Strains used in this study. Supplementary Table S2: Primers used in this study. Supplementary data: Full-length blots of the Western blot results.

## Abbreviations

The following abbreviations are used in this manuscript:

FLC	Fluconazole
ER	Endoplasmic reticulum
FAD	Flavin adenosine dinucleotide
NADPH	Nicotinamide adenine dinucleotide phosphate
SRE	Sterol regulatory elements
ERAD	Endoplasmic reticulum-associated degradation
MIC	Minimum inhibitory concentration
SMG	Super-MIC growth
GFP	Green fluorescent protein
CHX	Cycloheximide
m6A	N6-methyladenosine
AREs	AU-rich elements
UTR	Untranslated region
DMSO	Dimethyl sulfoxide
PBS	Phosphate-buffered saline
OD600	Optical density measurements at 600 nm
EDTA	Ethylene diamine tetraacetic acid
PMSF	Phenylmethylsulfonyl fluoride
SDS	Sodium dodecyl sulfate
PAGE	Polyacrylamide gel electrophoresis
PVDF	Polyvinylidene difluoride
SEM	Standard error of the mean
ANOVA	Analysis of variance
